# Effect of eardrum perforation and chronic otitis media on the results of infrared tympanic thermometer in adults: A systematic review and meta-analysis

**DOI:** 10.1097/MD.0000000000035932

**Published:** 2023-11-10

**Authors:** Yee-Hyuk Kim, Hee-Jun Park, Jae-Ho Yoo

**Affiliations:** a Department of Otorhinolaryngology-Head & Neck Surgery, Daegu Catholic University School of Medicine, Daegu, Korea; b Department of Otorhinolaryngology-Head & Neck Surgery, Daegu Catholic University Medical Center, Daegu, Korea.

**Keywords:** infrared, meta-analysis, temperature, thermometer, tympanic membrane perforation

## Abstract

**Background:**

This study was conducted to determine whether tympanic membrane perforation or chronic otitis media affects the results of an infrared tympanic membrane thermometer in adults.

**Methods:**

A literature search was performed using PubMed, Embase, Cochrane Library, Web of Science, and Google Scholar.

**Results:**

Four nonrandomized studies were included in the analysis. The temperatures of the bilateral eardrums (one eardrum with normal condition [control group] and the other eardrum with perforation or chronic otitis media [experimental group]) were measured for the same subject in the studies. The mean and standard deviation of the bilateral tympanic membrane temperatures were used to calculate the mean difference (MD) with a corresponding 95% confidence interval (CI). The fixed-effect model was utilized based on the results of the heterogeneity measurement using the Chi^2^ test and *I*^2^ statistic. The results of a meta-analysis in the normal eardrum (control group) and perforated eardrum, chronic suppurative otitis media with tympanic membrane perforation, or chronic otitis media with cholesteatoma (experimental group) were 343 subjects (MD = 0.05; 95% CI = −0.00 to 0.11; *P* = .06). A meta-analysis of the normal eardrum (control group) and perforated eardrum or chronic suppurative otitis media with tympanic membrane perforation except for cholesteatoma (experimental group) found 296 subjects (MD = 0.05; 95% CI = −0.01 to 0.11; *P* = .10).

**Conclusion:**

When the temperatures of the bilateral eardrums were measured using an infrared tympanic membrane thermometer, no difference was observed between the eardrum with perforation or chronic otitis media and the normal eardrum.

## 1. Introduction

One of the critical information that reflects the patient’s physical condition is body temperature. Due to the Coronavirus disease 2019 (COVID-19) pandemic, body temperature measurement is recognized as more important than ever before, not only in the medical field but also in daily life. An infrared tympanic membrane thermometer (ITMT) measures the radiant heat emitted from the eardrum; such heat is considered as the body temperature. This device requires a short testing time and provides relatively highly accurate results. Furthermore, it does not cause discomfort to patients and can be conveniently used by medical staff, hence its wide application in clinical settings.^[1–5]^ However, when the temperature is measured using an ITMT, the eardrum condition is not checked and is assumed to be normal. This study aimed to determine whether tympanic membrane perforation affects eardrum temperature measured using ITMT through a systematic review and meta-analysis. Suppose that eardrum perforation affects the result of the ITMT measurement, the ear thermometer reading, which is considered the body temperature, may be higher or lower than the actual value when the eardrum is perforated. As this can lead to errors in the assessment of the patient’s physical condition, it is crucial to determine whether a difference exists in the temperatures between a normal and a perforated eardrum.

## 2. Methods

### 2.1. Search strategy

Systematic literature review and meta-analysis were conducted according to the Preferred Reporting Items for Systemic Review and Meta-analyses guidelines.^[6]^ A literature search was performed using PubMed, Embase, Cochrane Library, Web of Science, and Google Scholar from the date of inception to February 06, 2023. The search terms are presented in the supplemental content (see Table S1, Supplemental Digital Content, http://links.lww.com/MD/K553, which shows the search strategy and result in each database), and the language was restricted to English.

### 2.2. Inclusion and exclusion criteria

Studies that compared the subjects’ bilateral eardrum temperatures measured using an ITMT for adults were included. Thus, all subjects had 1 normal eardrum and 1 with perforation or chronic otitis media. Studies that involved subjects with acute otitis media, otitis media with effusion, or eardrums with inserted ventilation tubes were excluded, as well as studies that included children or animals.

### 2.3. Data extraction and quality assessment

The primary outcome of this study was the difference in the temperatures between a normal eardrum and an eardrum with perforation or chronic otitis media measured using the ITMT.

In other words, the control group was the temperature of the normal eardrum, and the experimental group was the temperature of the opposite eardrum with perforation of the same subject. The difference in the temperatures between the bilateral eardrums was analyzed using the averages and standard deviations of both groups.

The 3 authors of this study independently reviewed each of the included studies. They extracted data on the first author’s name, year of publication, study location, study design, condition of the subjects’ eardrums, number and age of the subjects, and mean and standard deviation of the eardrum temperatures. In the case of inconsistent data, the reviewers resolved them through a discussion.

The patients included in the study by Kim (2022) had chronic suppurative otitis media (CSOM) with tympanic membrane perforation (n = 145) or chronic otitis media with cholesteatoma (n = 47).^[7]^ The patients’ tympanic membrane temperatures were measured the day before surgery. Among them, if only patients with CSOM with tympanic membrane perforation can be included in this study, it will be more similar to the subjects in Schmal (2006), Tasli (2018), and Cengiz (2021).^[8–10]^ Because Kim (2022) is one (Kim YH) of the authors of this study, analysis using bilateral tympanic membrane temperature results on the day before surgery for only patients with CSOM with tympanic membrane perforation could be separately implemented, excluding patients with chronic otitis media with cholesteatoma, which are the unpublished data of Kim (2022).

Among the data of Kim (2022), the published data (CSOM with tympanic membrane perforation and chronic otitis media with cholesteatoma) were summarized as Data (A) and the unpublished data (only CSOM with tympanic membrane perforation except for chronic otitis media with cholesteatoma) as Data (B).

The Risk of Bias Assessment Tool for Nonrandomized Studies (RoBANS) was used for the quality assessment of nonrandomized studies.^[11]^

### 2.4. Statistical analyses

To determine the effect of tympanic membrane perforation on the ITMT measurements, the temperature readings (mean ± standard deviation) in the normal and perforated eardrums were compared. Statistical analysis was conducted using Cochrane Review Manager (RevMan, version 5.4.1). The mean difference (MD) was calculated with 95% confidence intervals (CIs) for outcomes measured on a continuous scale. *P* < .05 was considered to indicate statistical significance. *P* < .1 in the Chi^2^ test or *I*^2^ > 50% in the *I*^2^ statistic was considered to denote statistically significant heterogeneity, and a random-effects model would be used, otherwise, a fixed-effects model.

### 2.5. Ethical consideration

Generally, a systematic review and a meta-analysis are exempt from Institutional Review Board screening due to the use of published data and noninclusion of individualized patient data. However, because this study included unpublished data from Kim (2022), approval from the Institutional Review Board was obtained (CR-23-034).

## 3. Results

### 3.1. Systematic literature review

**Figure 1.**
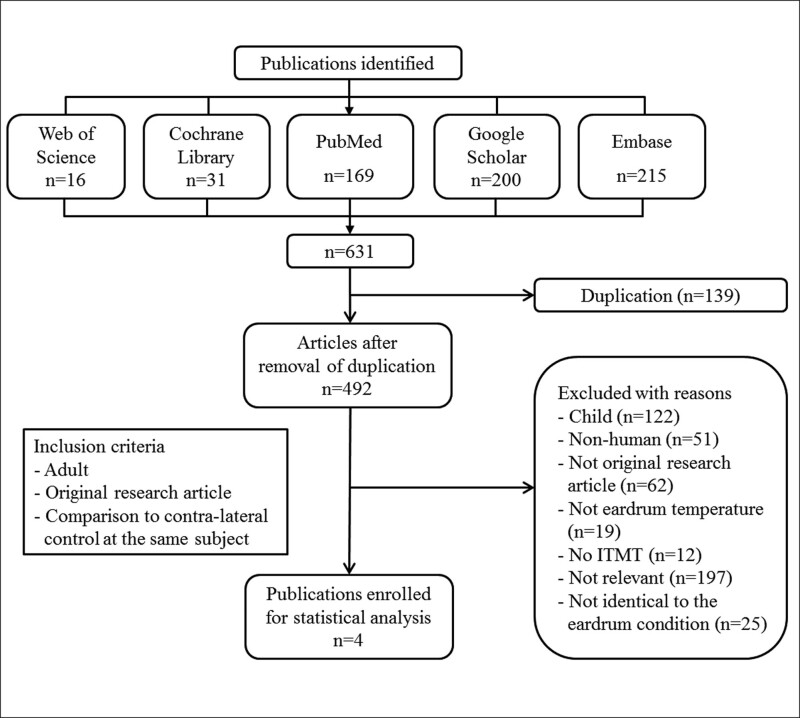
Preferred Reporting Items for Systematic Reviews and Meta-Analyses (PRISMA) flow diagram of the study selection process.

Through the literature search, 631 potentially relevant studies were initially identified, of which 4 were finally included in the analysis.^[7–10]^ The selection process for the studies to be included is presented in Figure 1.

### 3.2. Characteristics of the studies and quality assessment

Details of the 4 included studies and bilateral eardrum temperatures (control group, normal eardrum; experimental group, tympanic membrane contralateral to the normal eardrum in the same subjects) are presented in Table 1. The total number of subjects included in the 4 studies was 343. As mentioned in the Methods section, the results from the study by Kim (2022) are presented separately in Data (A) and Data (B).^[7–10]^

**Table 1:** Patient characteristics and summarized data of the included studies.

Year/author	Country	Design of the study	Otologic condition	No.	Age (mean ± SD) (yr)	Temperature (mean ± SD) (celsius)	*P* value
2022 Kim	South Korea	Data (A) Comparison of bilateral eardrum temperatures: chronic otitis media on one side (with cholesteatoma) and normal eardrum on the contralateral side in the same subjects (only published data) Temperature measurement time: the day before surgery.	1. CSOM with tympanic membrane perforation 2. Chronic otitis media with cholesteatoma	192	55.4 ± 11.31 (range: 22–79)	Experimental 37.09 ± 0.325	<.05
						Control 37.03 ± 0.330	
		Data (B) Comparison of bilateral eardrum temperatures: chronic otitis media with perforation on one side (without cholesteatoma) and normal eardrum on the contralateral side (including unpublished data) Temperature measurement time: the day before surgery.	CSOM with tympanic membrane perforation but without cholesteatoma	145	55.8 ± 11.72 (range: 22–79)	Experimental 37.08 ± 0.326	<.05
						Control 37.02 ± 0.334	
2021 Cengiz	Turkey	Comparison of the bilateral eardrum temperatures: perforated eardrum on one side and normal eardrum on the other side in the same subject.	Monaural dry perforation without drainage for the preceding 3 months and normal middle ear mucosa in patients with chronic otitis media	30	45.3 (range: 18–65) (SD: unknown)	Experimental 36.87 ± 0.51	≥.05
						Control 36.84 ± 0.51	
2018 Tasli	Turkey	Same as above	Monaural central perforation of the tympanic membrane without signs of infection	90	30.74 ± 9.61 (range: 20–58)	Experimental 36.34 ± 0.61	≥.05
						Control 36.33 ± 0.60	
2006 Schmal	Germany	Same as above	Same as above	31	40.0 ± 14.0 (range: unknown) (subject: adult patients)	Experimental 36.39 ± 0.28	≥.05
						Control 36.34 ± 0.24	

Author = the first author of each paper. CSOM: chronic suppurative otitis media. No.: number of subjects. Year: the publication year of each paper.

The RoBANS, which consisted of 6 domains, was used to assess the risk of bias in the included studies, and the results are presented in Table 2. Each domain of the RoBANS was evaluated according to the criteria for judgment in the Appendix section of the article.^[11]^ In the evaluation, the same results were obtained for all the included studies. “Selection of participants” was considered to be of low risk because the eardrum temperature was measured by separating both ears into a control group and an experimental group on the same subject. In the analysis, comparisons were made without stratification or statistical adjustment; thus, “Confounding variables” were considered to be of high risk. Because they included prospective or retrospective studies using tympanic temperature and findings in the tympanic membrane obtained from medical records, “Measurement of exposure” was considered to be of low risk. Although blinding was not performed, “Blinding of outcome assessments” could also be considered to be of low risk as blinding did not affect the temperature objectively measured using an ITMT. Because the data used for the analysis of the results were found to have no missing data, “Incomplete outcome data” was also considered to be of low risk. Furthermore, because primary outcomes were planned and analyzed in the research protocol, “Selective outcome reporting” was considered to be of low risk.

**Table 2:** Risk of bias assessment of nonrandomized using the RoBANS.

	Selection of participants	Confounding variables	Measurement of exposure	Blinding of outcome assessments	Incomplete outcome data	Selective outcome reporting
Kim 2022	Low	High	Low	Low	Low	Low
Cengiz 2021	Low	High	Low	Low	Low	Low
Tasli 2018	Low	High	Low	Low	Low	Low
Schmal 2006	Low	High	Low	Low	Low	Low

Risk of bias assessment was performed using the RoBANS for nonrandomized studies. Each domain was classified as either unclear risk, low risk, or high risk.

RoBANS: Risk of Bias Assessment Tool for Nonrandomized Studies.

### 3.3. Outcomes

**Figure 2.**
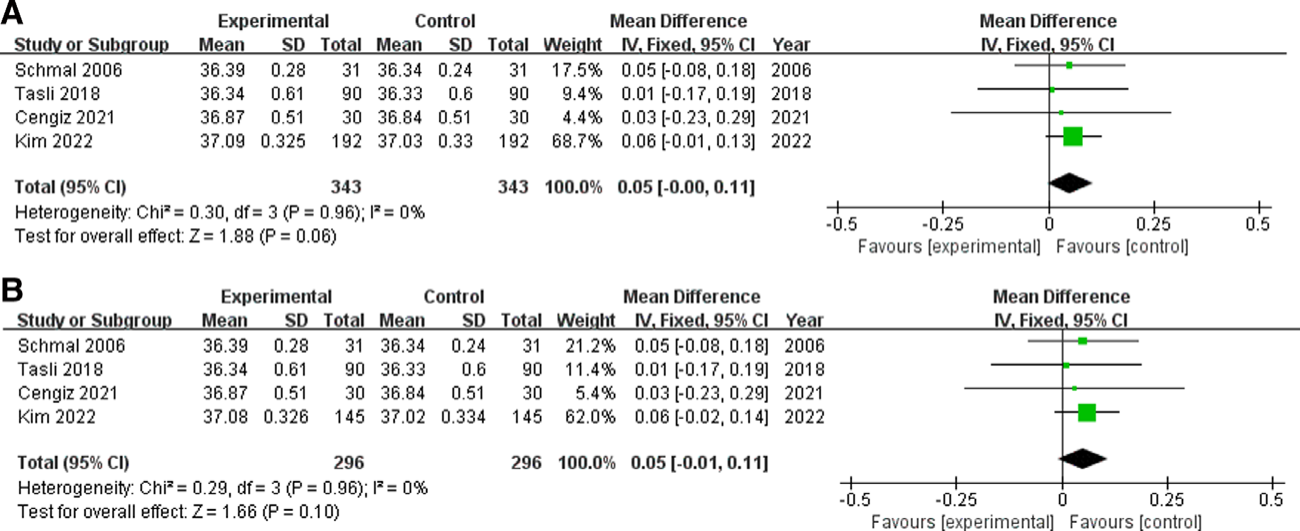
(A) Forest plot of Schmal (2006), Tasli (2018), Cengiz (2021), and Kim (2022) (Data [A]) (343 subjects in total). (B) Forest plot of Schmal (2006), Tasli (2018), Cengiz (2021), and Kim (2022) (Data [B]) (296 subjects in total).

The results of the 4 studies were analyzed for the difference in the temperatures between the normal and the perforated eardrums. In all these studies, the temperature of the tympanic membrane with perforation or chronic otitis media was higher than that of the normal eardrum. However, no statistically significant difference was observed in the fixed-effects model of the present study (Fig. 2).

When the MD in the temperatures between the bilateral eardrums was analyzed using Schmal (2006), Tasli (2018), Cengiz (2021), and Data (A) of Kim (2022) (343 subjects in total), the following result was obtained: MD = 0.05; 95% CI = −0.00 to 0.11; *P* = .06 (Fig. 2A). Furthermore, in the analysis using Schmal (2006), Tasli (2018), Cengiz (2021), and Data (B) of Kim (2022) (total subjects of 296), the following result was obtained: MD = 0.05; 95% CI = −0.01 to 0.11; *P* = .10 (Fig. 2B).

Both analysis results indicated that there was no difference in the temperatures between the normal eardrum and the eardrum with tympanic membrane with perforation or chronic otitis media.

### 3.4. Sensitivity analysis and publication bias

**Figure 3.**
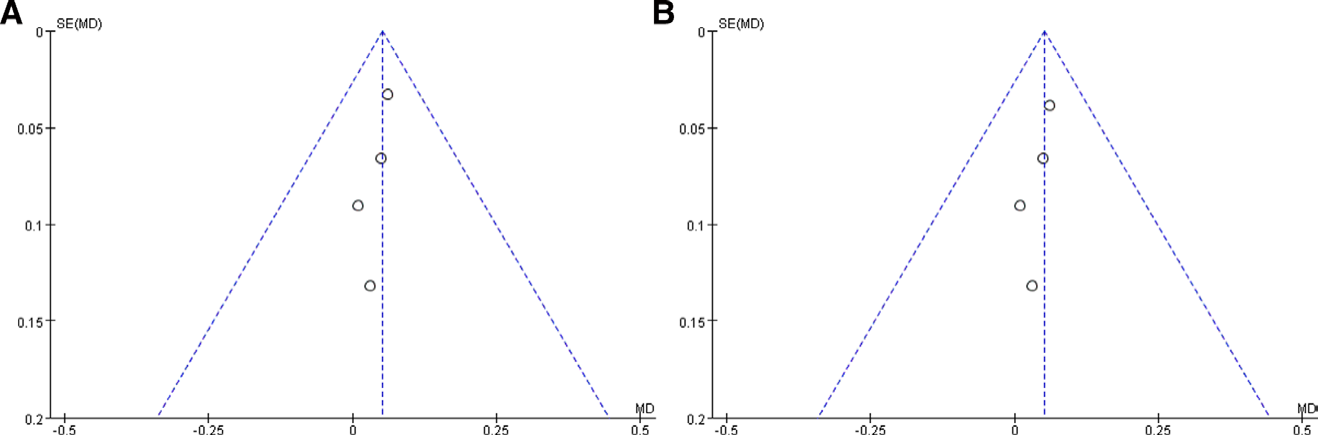
(A) Funnel plot of Schmal (2006), Tasli (2018), Cengiz (2021), and Kim (2022) (Data [A]). (B) Funnel plot of Schmal (2006), Tasli (2018), Cengiz (2021), and Kim (2022) (Data [B]).

In each analysis, which included Data (A) and Data (B) of Kim (2022), all of both *P* = .96 on the Chi^2^ test and *I*^2^ = 0% on the *I*^2^ statistic. Thus, a fixed-effects model was used in both analyses as significant heterogeneity was not observed among the studies (Fig. 2). Furthermore, a funnel plot was used to confirm publication bias. Although the number of studies included in the analysis was too small to analyze publication bias, it seemed that no apparent bias existed (Fig. 3).

## 4. Discussion

Chronic otitis media includes cases associated with middle ear cholesteatoma and CSOM with tympanic membrane perforation but no cholesteatoma. Kim (2022) presented the analysis results using eardrum temperatures measured the day before surgery in patients with chronic otitis media (CSOM with tympanic membrane perforation and chronic otitis media with cholesteatoma).^[7]^ The eardrum condition of the subjects in the studies by Schmal (2006) and Tasli (2018) was described as monaural central perforation of the tympanic membrane without signs of infection.^[8,9]^ “No signs of infection” can be interpreted as the absence of acute infection, which means there is no ear discharge. That is, the eardrum in the experimental group had dry perforation. Furthermore, the otologic condition of the subjects in the study by Cengiz (2021) was described as monaural dry perforation without drainage for the preceding 3 months and normal middle ear mucosa.^[10]^ The eardrum perforations were caused by chronic otitis media without cholesteatoma. It is necessary to note “CSOM with tympanic membrane perforation” among the subjects in the study by Kim (2022). The disease is usually operated on when the period without ear discharge lasts for at least 3 to 4 weeks.^[12]^ In other words, the tympanic membranes of the subjects had dry perforation during the temperature measurement. Because the tympanic membrane temperature was measured on the day before surgery, it can be assumed that the subjects’ eardrum condition was almost the same as that of the subjects included in the studies by Schmal (2006), Tasli (2018), and Cengiz (2021).

The experimental groups in the studies by Schmal (2006) and Tasli (2018) had perforation, the cause of which was not described. The most frequent cause of tympanic membrane perforation is chronic otitis media; another cause is trauma.^[13,14]^ Even if tympanic membrane perforation is caused by other than chronic otitis media, while the perforation of the eardrum persists, chronic otitis media is more likely to be accompanied. This may be because the perforated eardrum causes the exposed mucosa of the middle ear cavity to become vulnerable to infection more than the normal eardrum. Therefore, it is very likely that most of the subjects included in the studies by Schmal (2006) and Tasli (2018) had eardrum perforation (eardrum perforation caused by chronic otitis media or accompanying chronic otitis media after eardrum perforation) associated with chronic otitis media. In summary, the analysis using Data (A) of Kim (2022) is close to the result of the effect of chronic otitis media on temperature measurement using ITMT; the result of the analysis using Data (B) of Kim (2022) can be considered as a result of analyzing the effect of the perforated eardrum on temperature measurement using ITMT.

The ITMT measures the radiant heat emitted from the tympanic membrane.^[15,16]^ If the eardrum is perforated, it can be considered that the infrared radiation released from not only the eardrum but also the mucosal membrane of the middle ear cavity can be detected by the ITMT sensor. This study aimed to determine whether such a situation affects the temperature measurement using ITMT through a systematic review and meta-analysis. When the results of individual studies published so far, Schmal (2006), Tasli (2018), and Cengiz (2021), no statistically significant difference was observed in the temperatures between the normal and perforated eardrums; on the other hand, Data (A) and Data (B) of Kim (2022) indicated statistically significant differences. Therefore, the statistical analyses of the individual studies did not reveal consistent results. Because the tympanic membrane and hypothalamus share arterial blood supply from the carotid artery, the eardrum temperature is thought to be a direct reflection of the core temperature.^[15–18]^ Considering that the middle ear cavity receives the same arterial blood supply from the carotid artery as the tympanic membrane,^[18]^ it can be inferred that no difference exists in the temperatures between the perforated and the normal eardrums. However, because the distance from the exposed mucosa of the middle ear cavity to the ITMT sensor is greater than that from the eardrum to it, the temperature of the perforated eardrum could be expected to be lower than that of the normal eardrum. Conversely, because the mucosa of the middle ear cavity is located more internally than the eardrum and is less affected by outside temperature, the ITMT readings could be expected to be higher in the perforated eardrum than in the normal eardrum. In this study, no difference was observed in the temperatures between the eardrum with perforation or chronic otitis media and the normal eardrum measured using ITMT.

## 5. Conclusion

A meta-analysis was conducted using data from 4 nonrandomized studies. It was found that tympanic membrane perforation or chronic otitis media without acute inflammation did not affect the temperature measurement using ITMT in adults.

## Author contributions

**Conceptualization:** Yee-Hyuk Kim.

**Data curation:** Yee-Hyuk Kim, Hee-Jun Park, Jae-Ho Yoo.

**Formal analysis:** Yee-Hyuk Kim.

**Methodology:** Yee-Hyuk Kim, Hee-Jun Park, Jae-Ho Yoo.

**Writing – original draft:** Yee-Hyuk Kim.

**Writing – review & editing:** Yee-Hyuk Kim, Hee-Jun Park, Jae-Ho Yoo.

## Supplementary Material


