# Supplementary material for: Effect of eardrum perforation and chronic otitis media on the results of infrared tympanic thermometer in adults: A systematic review and meta-analysis
Source: Medicine (Baltimore). 2023 Nov 10;102(45):e35932. doi: 10.1097/MD.0000000000035932 (PMC10637521; doi:10.1097/MD.0000000000035932)
Supplement: Supplementary file 1 [file medi-102-e35932-s001.docx]

Supplemental content for search strategy and result in each database

1. Search strategy in PubMed

| Search query | | Items found |
| --- | --- | --- |
| #1 | "infrared"[All Fields] OR "ear"[MeSH Terms] OR "ear"[All Fields] OR "tympanal"[All Fields] OR "tympanate"[All Fields] OR "tympanic"[All Fields] | 445,747 |
| #2 | (thermomet* [mesh]) OR (thermomet* [tw]) | 19,031 |
| #3 | temperature | 1,031,193 |
| #4 | ((ear) OR (eardrum) OR (tympanic) OR (tympanic membrane)) AND (perforat*) | 4,996 |
| #5 | (otitis) AND (media) | 31,774 |
| #6 | #1 AND #2 | 3,937 |
| #7 | #1 AND #3 | 46,232 |
| #8 | #4 OR #5 | 35,112 |
| #9 | #6 OR #7 | 46,961 |
| #10 | #8 AND #9 | 169 |

2. Search strategy in Embase

| Search query | | Items found |
| --- | --- | --- |
| #1 | ('infrared'/exp OR infrared) AND thermomet* | 2,270 |
| #2 | (‘ear’/exp OR ear OR ‘eardrum’/exp OR eardrum OR tympanic) AND (‘temperature’/exp OR temperature) | 5,994 |
| #3 | ‘eardrum’/exp OR eardrum | 14,208 |
| #4 | ‘tympanic membrane’/exp OR ‘tympanic membrane’ OR (tympanic AND (‘membrane’/exp OR membrane)) | 14,743 |
| #5 | perforat* | 177,965 |
| #6 | ‘otitis media’/exp OR ‘otitis media’ OR ((‘otitis’/exp OR otitis) AND (‘media’/exp OR media)) | 47,087 |
| #7 | #1 OR #2 | 7,811 |
| #8 | #3 AND #5 | 4,892 |
| #9 | #4 AND #5 | 3,766 |
| #10 | #6 OR #8 OR #9 | 50,268 |
| #11 | #7 AND #10 | 215 |

3. Search strategy in Google scholar, Web of Science, and Cochrane library

| Search engine | Boolean operators | Items found |
| --- | --- | --- |
| Google scholar | ((((infrared) AND (thermomet*)) OR ((infrared) AND (temperature))) AND (((ear) OR (eardrum)) OR (tympanic membrane))) AND (((otitis media) OR ((eardrum) AND (perforat*))) OR ((tympanic membrane) AND (perforat*))) | 200 |
| Web of Science | ((((ear) OR (eardrum) OR (tympanic) OR (tympanic membrane)) AND (perforat*)) OR ((otitis) AND (media))) AND ((((infrared) OR (ear) OR (tympanic)) AND (thermomet*)) OR (((infrared) OR (ear) OR (tympanic)) AND (temperature))) | 16 |
| Cochrane library |  | 31 |
